# Treatment with a Catalytic Superoxide Dismutase (SOD) Mimetic Improves Liver Steatosis, Insulin Sensitivity, and Inflammation in Obesity-Induced Type 2 Diabetes

**DOI:** 10.3390/antiox6040085

**Published:** 2017-11-01

**Authors:** Gina M. Coudriet, Meghan M. Delmastro-Greenwood, Dana M. Previte, Meghan L. Marré, Erin C. O’Connor, Elizabeth A. Novak, Garret Vincent, Kevin P. Mollen, Sojin Lee, H. Henry Dong, Jon D. Piganelli

**Affiliations:** 1 Department of Surgery, Children’s Hospital of Pittsburgh of UPMC, School of Medicine, University of Pittsburgh, Pittsburgh, PA 15224, USA; gmr9@pitt.edu (G.M.C.); dmp51@pitt.edu (D.M.P.); mem212@pitt.edu (M.L.M.); eco9@pitt.edu (E.C.O.); elizabeth.novak@chp.edu (E.A.N.); gav10@pitt.edu (G.V.); kevin.mollen@chp.edu (K.P.M.); 2 Department of Pediatrics, Children’s Hospital of Pittsburgh of UPMC, School of Medicine, University of Pittsburgh, Pittsburgh, PA 15224, USA; meghan.delmastro@gmail.com (M.M.D.-G.); sol24@pitt.edu (S.L.); dongh@pitt.edu (H.H.D.)

**Keywords:** SOD mimetic, metalloporphyrin, inflammation, type 2 diabetes, NAFLD, obesity, insulin resistance

## Abstract

Oxidative stress and persistent inflammation are exaggerated through chronic over-nutrition and a sedentary lifestyle, resulting in insulin resistance. In type 2 diabetes (T2D), impaired insulin signaling leads to hyperglycemia and long-term complications, including metabolic liver dysfunction, resulting in non-alcoholic fatty liver disease (NAFLD). The manganese metalloporphyrin superoxide dismustase (SOD) mimetic, manganese (III) meso-tetrakis (*N*-ethylpyridinium-2-yl) porphyrin (MnP), is an oxidoreductase known to scavenge reactive oxygen species (ROS) and decrease pro-inflammatory cytokine production, by inhibiting nuclear factor kappa-light-chain-enhancer of activated B cells (NF-κB) activation. We hypothesized that targeting oxidative stress-induced inflammation with MnP would assuage liver complications and enhance insulin sensitivity and glucose tolerance in a high-fat diet (HFD)-induced mouse model of T2D. During 12 weeks of feeding, we saw significant improvements in weight, hepatic steatosis, and biomarkers of liver dysfunction with redox modulation by MnP treatment in HFD-fed mice. Additionally, MnP treatment improved insulin sensitivity and glucose tolerance, while reducing serum insulin and leptin levels. We attribute these effects to redox modulation and inhibition of hepatic NF-κB activation, resulting in diminished ROS and pro-inflammatory cytokine production. This study highlights the importance of controlling oxidative stress and secondary inflammation in obesity-mediated insulin resistance and T2D. Our data confirm the role of NF-κB-mediated inflammation in the development of T2D, and demonstrate the efficacy of MnP in preventing the progression to disease by specifically improving liver pathology and hepatic insulin resistance in obesity.

## 1. Introduction

The prevalence of obesity continues to be a worldwide concern, and in the United States, roughly one-third of the American adult population is considered to be obese [1]. Over-nutrition, a sedentary lifestyle, and genetic predisposition are strongly associated with the development of insulin resistance and metabolic syndrome, both of which are major risk factors for developing type 2 diabetes (T2D) [2,3]. Increased central adiposity causes peripheral organs, like the liver, to become targets of ectopic fat deposition, which can lead to non-alcoholic fatty liver disease (NAFLD), the most common liver disorder in the Western world [4,5]. Consequently, over 90% of obese patients diagnosed with T2D also have NAFLD [6], a condition that presents with hepatic insulin resistance [7,8].

The prevailing paradigm is that persistent adiposity, both central and peripheral, induces oxidative stress and chronic inflammation [9,10,11], causing insulin resistance [12]. Inflammation and more specifically, obesity-induced inflammation is controlled by the redox-dependent transcription factor, NF-κB, which is elevated in many tissues in models of obesity [13,14]. Circulating levels of NF-κB-dependent pro-inflammatory cytokines, chemokines, and acute phase proteins are also detected in T2D [15]. Increased NF-κB activity and the ensuing inflammation contribute to the dysregulation of insulin signaling at peripheral sites, like the liver [9,13,14,16], perpetuating NAFLD. As a result of chronic inflammation, global insulin receptor signaling becomes desensitized, resulting in impaired glucose metabolism [12,17,18].

Therefore, it is clear that persistent obesity-induced inflammation and oxidative stress can impact the progression to NAFLD, insulin resistance, and resultant T2D. The manganese porphyrin superoxide dismutase (SOD) mimetic, manganese (III) meso-tetrakis (*N*-ethylpyridinium-2-yl) porphyrin (MnP) is a first-in-class oxidoreductase that not only scavenges reactive oxygen species (ROS), like superoxide, but also possesses anti-inflammatory properties [19] and has been proven clinically safe [20]. MnP has been previously described as having positive effects in inflammatory-mediated disease models [21,22,23,24,25,26,27,28]. Work from our laboratory demonstrated that MnP inhibits inflammation by oxidation of the NF-κB p50 subunit, thus impeding DNA-binding and in turn, transactivation [22]. Additionally, the ability to scavenge superoxide by MnP results in the production of hydrogen peroxide, further contributing to oxidation of NF-κB p50 [29]. Taken together, we hypothesized that MnP would be effective in inhibiting obesity-induced NF-κB driven inflammation in the livers of obese mice, thus maintaining glucose homeostasis and promoting insulin sensitivity, while diminishing NAFLD.

## 2. Materials and Methods

### 2.1. Cell Culture

The human hepatocellular carcinoma cell line, HepG2 (American Type Culture Collection (ATCC), Manassas, VA, USA), was a generous gift from H. Henry Dong (University of Pittsburgh). The cells were cultured in Eagle’s Minimum Essential Medium supplemented with 10% fetal bovine serum in a 37 °C in a 5% CO_2_ humid air incubator.

### 2.2. Preparation of Palmitic Acid (PA) Solution

PA solution was prepared by conjugating the fatty acid (FA) with FA-free bovine serum albumin (BSA; Sigma-Aldrich, St. Louis, MO, USA), as previously described [30]. Briefly, PA was dissolved in preheated 0.1 N NaOH and diluted in prewarmed serum-free media, containing 12% (*w*/*v*) BSA, to give a final FA concentration of 4.0 mM. Stock solution was sterile filtered. 

### 2.3. Measurement of Superoxide by Dihydroethidium (DHE)

HepG2 cells were stimulated with 100 µM PA, with or without 68 µM MnP, for 24 h in serum free media. Superoxide was measured using DHE (Molecular Probes, Waltham, MA, USA) by flow cytometry as previously described [28,31]. DHE measures total superoxide production, specifically, by the detection of 2-OH-ethidium by flow cytometry [31]. Briefly, after stimulation, cells were washed with ice cold phosphate buffered saline (PBS) and loaded with 50 µM DHE for 20 min at 37 °C. Mean fluorescence intensity (MFI) was read by a BD LSR II Flow Cytometer (BD Bioscience, San Jose, CA, USA) in the AmCyan channel using a 585/42 detector and 545LP filter [31]. DHE MFI is displayed as normalized to untreated DHE loaded cells.

### 2.4. Mice and Treatment

C57BL/6J mice (Jackson Labs, Bar Harbor, ME, USA) were housed under specific pathogen-free conditions in the Rangos Research Center at Children’s Hospital of Pittsburgh of UPMC. Animal experiments were approved by the Institutional Animal Care and Use Committee of Children’s Hospital of Pittsburgh (Assurance Number A3187-01) and were in compliance with the laws of the United States of America. Male mice (6–8 weeks of age) were fed either standard chow diet or a high fat diet (HFD) (Research Diets, Inc, New Brunswick, NJ, USA) consisting of 60% kcal fat for 12 weeks (*n* = 5 per group). MnP, a generous gift from James Crapo, MD (National Jewish Health), was prepared [21] and used at 5 mg/kg subcutaneously every 3 days.

### 2.5. Body Weight and Liver Weight

Body weight was recorded weekly up until termination of the experiment. At the time of sacrifice, whole livers were excised from individual animals, weighed, and photographed.

### 2.6. Comprehensive Diagnostic Panel

Blood samples from lean or HFD + MnP mice were collected for toxicity profiling using a VetScan Comprehensive Diagnostic Profile with a VetScan Chemistry Analyzer (Abaxis, Union City, CA, USA). Liver, kidney, and pancreatic dysfunction were tested.

### 2.7. Quantification of Hepatic Lipid Accumulation

At sacrifice, livers were excised, immediately snap frozen in liquid nitrogen, and stored at −80 °C. Hepatic intracellular triglyceride (TG) content was determined by acetone extraction as previously described and levels are defined as mg TG per gram of total liver protein [32].

### 2.8. Liver Histology

Liver tissue was harvested, fixed in 10% formalin, and embedded in paraffin, or incubated in 30% sucrose and embedded in Tissue-Tek optimum cutting temperature (O.C.T.) Compound (Sakura Finetek USA, Inc., Torrence, CA, USA). Paraffin sections, cut by the University of Pittsburgh Histology Core Laboratory were stained with hematoxylin and eosin (H&E). Frozen sections were cut and stained with Oil Red O by the core as well. Sections were visualized via an Axioplan 2 microscope (Zeiss, Oberkochen, Germany). Staining was quantified using ImageJ Software (NIH, Bethesda, MD, USA).

### 2.9. 8-Hzydroxy-2’-deoxyguanosine (8-OHdG)

Paraffin sections were used for 8-OHdG immunofluorescence. Sections were deparaffinized, rehydrated, and citric acid was used for antigen retrieval. Sections were blocked with 5% donkey serum for 1 h at room temperature and then stained with the 8-OHdG primary antibody (Abcam, Cambridge, MA, USA) overnight at 4 °C. The following day, sections were probed with a donkey anti-mouse AlexaFluor555 tagged secondary antibody for 1 h at room temperature. Lastly, sections were stained with 4′,6-diamidino-2-phenylinodle (DAPI) and coverslips were mounted. Sections were imaged using an Olympus fluorescent microscope (Olympus, Center Valley, PA, USA). Three fields per section were imaged and staining was quantified using ImageJ Software (NIH, Bethesda, MD, USA) by a blinded researcher. Nuclei, positive for 8-OHdG staining, were divided by total viable nuclei to give a percentage of positively stained nuclei. 

### 2.10. Serum Adipokine Measurement

After overnight fasting, blood samples were collected and serum adipokines were determined by a Milliplex Mouse Serum Adipokine Panel (Millipore, Billerica, MA, USA), at 5, 10, and 12 weeks of feeding.

### 2.11. Intraperitoneal Glucose Tolerance Test (IPGTT)

A 1.0 g/kg dose of a 10% glucose solution was administered intraperitoneally after overnight fasting, after 10 weeks of HFD feeding. Blood samples were collected at 0, 30, 60, 90 and 120 min after glucose injection. Blood glucose levels were measured using a glucometer (Ascensia Breeze 2, Bayer, Leverkusen, Germany).

### 2.12. Insulin Tolerance Test (ITT)

A 0.75 U dose of insulin (HumulinR, Lilly USA, Indianapolis, IN, USA) was injected intraperitoneally, under non-fasting conditions, after 12 weeks of HFD feeding. Blood glucose levels were measured at 0, 15, 30, 60, 90, and 120 min, post-insulin injection, using a glucometer.

### 2.13. Homeostasic Model Assessment-Estimated Insulin Resistance (HOMA-IR)

Insulin sensitivity was estimated by the HOMA-IR index calculated by the following equation: HOMA-IR = fasting glucose (mg/dL) × fasting insulin (U/mL) ÷ 405 [32].

### 2.14. Nuclear Protein Extractions and Electrophoretic Mobility Shift Assay (EMSA)

Nuclear proteins were extracted from frozen livers as previously described [33]. The DNA-binding oligonucleotide and corresponding complementary strand for NF-κB, 5′-AGTTGAGGGGACTTTCCCAGGC-3′, (Promega, Madison, WI, USA), was end-labeled with ^32^P. Nuclear liver extract (36 μg), HeLa nuclear extract (positive control), and the cold competitor consisting of HeLa nuclear extract incubated with 50-fold excess of the unlabeled NF-κB consensus DNA-binding oligo were incubated with the ^32^P-labeled oligo. The samples were resolved in a 4% Acrylamide, 60:1 Acrylamide:Bisacrylamide gel. The gel was dried and exposed to film at −80 °C and then developed.

### 2.15. Quantitative Real-Time PCR (qRT-PCR)

RNA isolation from liver tissue was performed (RNeasy Kit, Qiagen, Valencia, CA, USA), and cDNA was synthesized (RT^2^ Frist Strand Kit Qiagen, Valencia, CA, USA). Gene-specific cDNA was amplified and quantified in a real-time thermal iCycler system (BioRad, Hercules, CA, USA) using SYBR Green all-in-one qPCR mix (GeneCopoeia, Rockville, MD, USA). The primer sequences encoding transcripts for mouse tumor necrosis factor-α (*tnfα*), macrophage chemoattractant protein-1 (*mcp1*), inducible nitric oxide synthase (*inos*), and 50S ribosomal protein L15 (*rplo*) were: *tnfα* (FWD: 5′-TTCCGAATTCACTGGAGCCTCGAA-3′, REV: 5′-TGCACCTCAGGGAAGAATCTGGAA-3′); *mcp1* (FWD: 5′-ATGCAGTTAACGCCCCACTC-3′, REV: 5′-CCCATTCCTTCTTGGGGTCA-3′); *inos* (FWD: 5′-CTGCTGGTGGTGACAAGCACATTT-3′, REV: 5′-ATGTCATGAGCAAAGGCGCAGAAC-3′); and *rplo* (FWD: 5′-GGCGACCTGGAAGTCCAACT-3′, REV: 5′-CCATCAGCACCACAGCCTTC-3′).

### 2.16. Enzyme-Linked Immunosorbent Assay (ELISA)

The hepatic MCP-1 and TNF-α protein concentrations were measured by ELISA (Abcam, Cambridge, MA, USA). Liver lysates were prepared using the Abcam supplied lysate buffer. Absorbance was read at 450 nm, and hepatic cytokine content was determined as pg of cytokine per mg of liver protein.

### 2.17. Statistical Analysis

Data were analyzed using GraphPad Prism 6.0 (GraphPad, La Jolla, CA, USA). When two variables were measured, data was analyzed by two-way analysis of variance (ANOVA) with a Tukey or Bonferroni post-hoc analysis for multiple comparisons. When one variable was analyzed, a one-way ANOVA with a Tukey post-test of multiple comparisons was used. When data from two groups was compared, an unpaired *t* test was used. Data are shown as mean ± standard error of the mean (SEM) or standard deviation (SD). A *p* value < 0.05 was considered statistically significant. All tests and statistical parameters are indicated in the figure legends.

## 3. Results

### 3.1. MnP Diminishes Superoxide Production In Vitro

**Figure 1:**
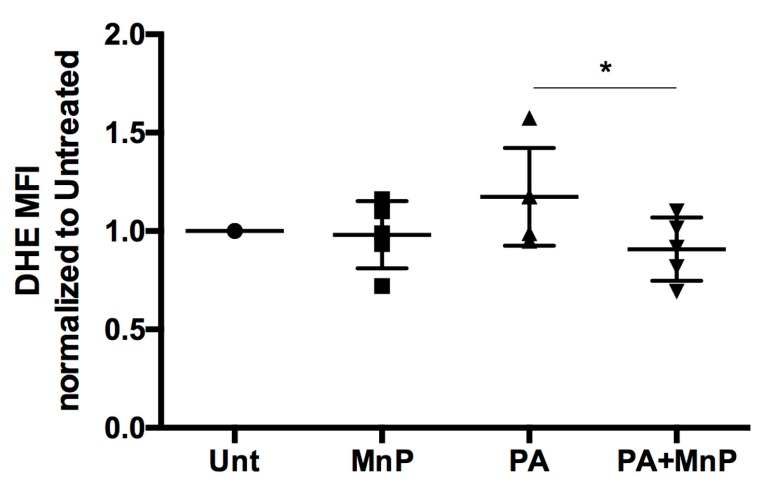
MnP diminishes superoxide production in vitro. HepG2 cells were treated with 100 µM of PA, with or without 68 µM MnP, for 24 h. Cells were then loaded with DHE and read for fluorescence by flow cytometry. Unt: untreated, MnP: 68 µM MnP alone, PA: 100 µM PA alone, PA + MnP: 100 µM PA + 68 µM MnP. Data are displayed as mean fluorescence intensity (MFI) normalized to untreated controls ± SEM. Significance was determined by *t*-test of a combined *n* = 5 experiments (* indicates *p* < 0.05).

Oxidative stress is the detrimental basis for many inflammatory-mediated disease pathologies [34]. Treatment for these diseases, like T2D, include antioxidants, to scavenge free radicals that are responsible for promoting inflammation [35,36]. Previous work from our group has demonstrated MnP as a potent SOD mimetic, with catalytic and anti-inflammatory properties. Its ability to scavenge superoxide in immune cells has been extensively shown [22,23,25,27,28]. MnP also protects pancreatic β cells from oxidative damage [24,37,38]. In order to demonstrate that MnP would be an effective hepatic antioxidant, we interrogated the human liver carcinoma cell line, HepG2, as a basis for our study on obesity-induced NAFLD and T2D. Stimulation of HepG2 cells in vitro with palmitic acid (PA) is a widely accepted experimental model of hepatocellular steatosis [39]. HepG2 cells possess nicotinamide adenine dinucleotide phosphate (NADPH) oxidase 3 (NOX3), responsible for the generation of ROS, like superoxide [40]. Therefore, we used this model of PA-induced superoxide production to demonstrate the ability of MnP to quell hepatic ROS. Our data show that HepG2 cells stimulated with 100 µM of PA for 24 h [40] led to an increase in superoxide production, as measured by DHE fluorescence (Figure 1). Treatment with 68 µM MnP was successful in scavenging superoxide, by significantly reducing (*p* < 0.05) its production by DHE MFI. Therefore, we propose that MnP would be an effective antioxidant in our in vivo model, capable of scavenging hepatic ROS and inhibiting resultant inflammation.

### 3.2. MnP Treatment Reduces Weight Gain and Imposes Minimal Toxicity

**Figure 2:**
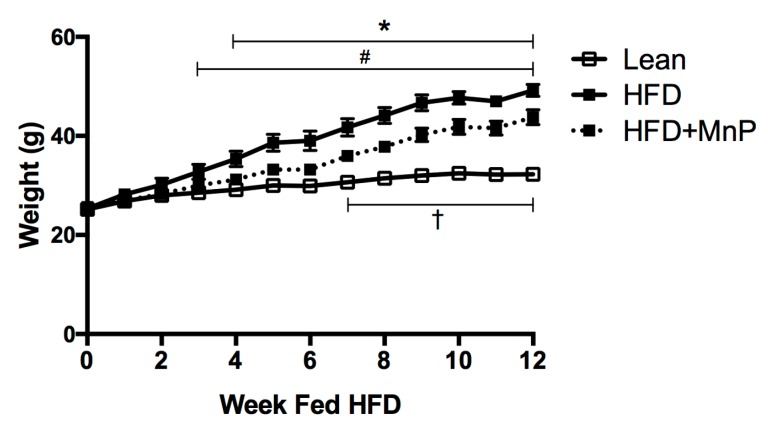
MnP treatment reduces weight gain in high fat diet (HFD)-fed mice. Over 12 weeks of HFD feeding, weights were recorded for Lean (empty squares, solid line), HFD (filled squares, solid line), and HFD + MnP (filled squares, dashed line) mice, *n* = 5 mice per group. Data is displayed as mean ± SEM. Significance was calculated by two-way ANOVA with a Tukey post-test of multiple comparisons. Significant differences were observed between Lean and HFD starting at 3 weeks (#), between HFD and HFD + MnP starting at 4 weeks (*), and between Lean and HFD + MnP starting at 7 weeks (†), all lasting the duration of the experiment.

The HFD-fed mouse has been extensively studied and is a well-accepted model for obesity-induced insulin resistance [41]. We employed this model to test our hypothesis that inhibition of inflammation by MnP improves glucose tolerance and insulin sensitivity in T2D. HFD-fed mice gained weight as expected; however, MnP treatment significantly reduced weight gain during HFD feeding (Figure 2). Lean mice gained significantly less weight than their HFD fed counterparts (*p* < 0.05) from 3 to 12 weeks. Significant differences in weight gain were detected between the HFD and HFD + MnP treated mice from 4 to 12 weeks (*p* < 0.05). Moreover, significant weight gain between lean and HFD + MnP mice was not observed until 7 weeks of feeding, lasting until 12 weeks (*p* < 0.05). These data indicate that onset of high calorie-induced weight gain is diminished upon redox modulation.

Previous work has proven that MnP treatment is well-tolerated as it has passed extensive safety-toxicology studies for parenteral therapy [20]. Specifically, in mice, the no observed adverse effect level (NOAEL) was established to be 10 mg/kg/day after once-daily iv dosing for 18 days [20]. Based on these findings, we did not anticipate any toxicity in our study, since only 5 mg/kg dose of MnP was administered, subcutaneously, every 3 days. In this study, to confirm that MnP treatment did not cause toxicity that contributed to the loss in weight gain (Figure 2), a comprehensive diagnostic profile was conducted, examining liver, kidney, and pancreatic dysfunction (Table 1). No significant toxicity was observed during this study and all values stayed within the normal clinical range [42]. Therefore, all observed improvements in weight, liver pathology, and diabetes in this study can be attributed to the redox modulation of MnP.

### 3.3. Redox Modulation Reduces Liver Steatosis and Hepatic Lipid Accumulation

**Figure 3:**
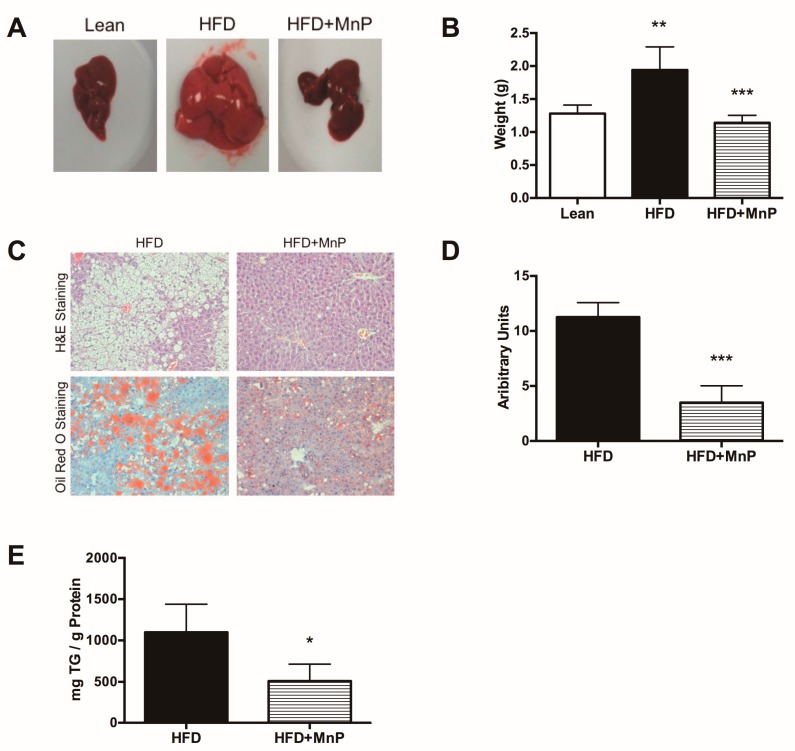
Redox modulation reduces liver steatosis and hepatic lipid accumulation in HFD-fed mice. (**A**) Gross examination of livers excised from lean mice and mice fed HFD for 12 weeks, with or without MnP treatment. Representative images are shown; (**B**) Weights of livers excised after 12 weeks of feeding. Data are displayed as mean ± SD, *n* = 5 per group. Significance was calculated by one-way ANOVA with a Tukey post-test of multiple comparisons. ** indicates *p* ≤ 0.01, *** indicates *p* ≤ 0.001; (**C**) H&E (top row) and Oil Red O (bottom row) staining of livers after 12 weeks of HFD feeding, with or without MnP treatment. Representative images are shown with a 20× magnification; (**D**) Quantification of Oil Red O staining. Data are displayed as mean ± SD, *n* = 4 mice per group. Three images per section were analyzed using Image J (NIH, Bethesda, MD, USA). Significance was calculated by *t*-tests. *** indicates *p* ≤ 0.001; (**E**) Hepatic intracellular triglyceride (TG) levels, defined as mg TG per gram of total liver protein, were measured by acetone extraction. Data are displayed as mean ± SD. *n* = 5 mice per group. Significance was calculated by *t*-tests. * indicates *p* ≤ 0.05.

In order to assess the degree of hepatic steatosis, mice were euthanized at 12 weeks and livers were excised immediately thereafter, to be photographed and weighed, after 12 weeks of HFD feeding. A clear difference in the size and degree of fat accumulation, based on color, was observed between the groups (Figure 3A). HFD-feeding significantly increased liver weight compared to lean chow-feeding (*p* ≤ 0.01), while MnP treatment significantly reduced liver weight (Figure 3B) (*p* ≤ 0.001), comparable to that of lean mice. Liver sections were assessed for fat droplets by H&E staining and Oil Red O staining (Figure 3C). Quantification of Oil Red O staining demonstrated that MnP treatment diminished fat accumulation in HFD livers compared to untreated HFD livers (Figure 3D) (*p* ≤ 0.001). Further, MnP treatment significantly reduced (*p* ≤ 0.05) the levels of intrahepatic triglycerides, as compared to HFD alone (Figure 3E). Taken together, these data demonstrate that redox modulation by MnP treatment reduces steatosis in HFD-induced obesity.

### 3.4. Hepatic Insulin Resistance is Improved with MnP Treatment

**Figure 4:**
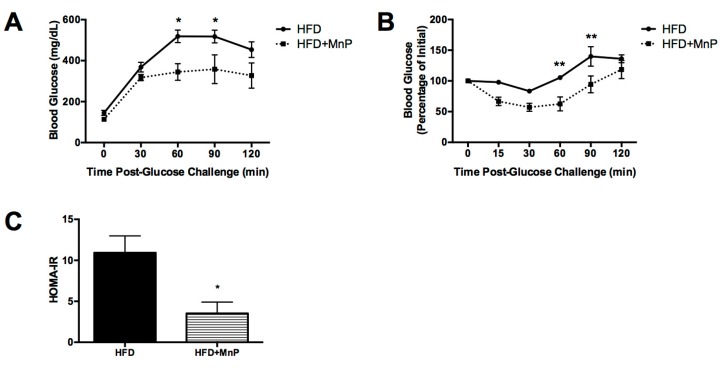
Hepatic insulin resistance is improved with MnP treatment. (**A**) After 10 weeks of HFD feeding, mice were fasted and subjected to an IPGTT. Data are displayed as mean ± SEM. Significance was calculated by two-way ANOVA with a Bonferroni post-test of multiple comparisons. * indicates *p* ≤ 0.05; (**B**) After 10 weeks of HFD feeding, mice were fasted and subjected to an ITT. Data displayed as mean ± SEM. Significance was calculated by two-way ANOVA with a Bonferroni post-test of multiple comparisons. ** indicates *p* ≤ 0.01; (**C**) HOMA-IR was calculated from these results. Data are displayed as mean ± SEM. Significance was calculated by *t*-tests. * indicates *p* ≤ 0.05.

After 10 weeks of HFD feeding, with and without MnP treatment, Intraperitoneal glucose tolerance test (IPGTT) and Insulin tolerance test (ITT) were performed, in order to assess glucose intolerance and insulin resistance. MnP treated animals exhibited significant improvements in glucose tolerance, 60 and 90 min post-glucose challenge, as compared to HFD mice (*p* ≤ 0.05) (Figure 4A), indicating enhanced glucose clearance. Improved glucose tolerance with MnP treatment also correlated with improved insulin sensitivity at the same time points (*p* ≤ 0.01) (Figure 4B). Further, an estimation of insulin sensitivity was calculated using HOMA-IR. The HOMA-IR score is derived from a calculation accounting for the presence and extent of insulin resistance [43]. A higher HOMA-IR score translates to increased insulin resistance. Here, the HOMA-IR score was significantly decreased (*p* ≤ 0.05) with MnP treatment compared to HFD alone (Figure 4C). These results indicate enhanced insulin sensitivity and improved glucose clearance due to MnP treatment, which can be attributed in part to the reduction in hepatocellular lipids exhibited by these animals (Figure 3).

### 3.5. Insulin, Leptin, and Plasminogen Activator Inhibitor Type-1 (PAI-1) Levels are Diminished Following MnP Treatment

Since MnP reduced liver steatosis (Figure 3) and maintained insulin sensitivity (Figure 4), we sought to determine whether MnP, in turn, would reduce the effects of these phenomena in our disease model. Specifically, we examined hyperinsulinemia, hyperleptinemia, and the cardiovascular risk factor, plasminogen activator inhibitor-1 (PAI-1).

**Figure 5:**
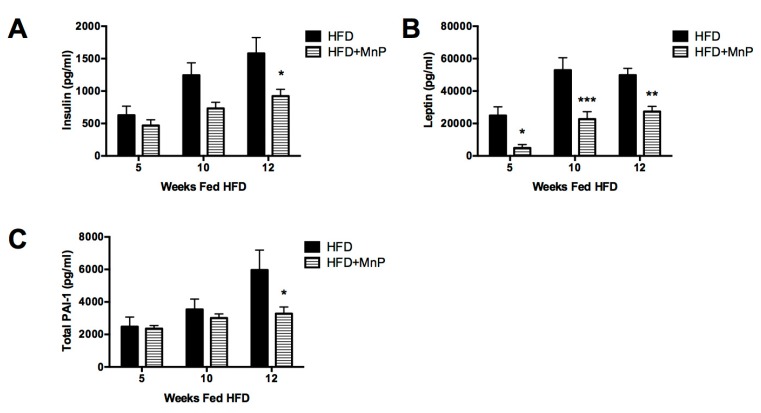
MnP treatment lowered serum adipokines. (**A**) serum insulin; (**B**) serum leptin and (**C**) serum PAI-1 were measured at 5, 10, and 12 weeks of HFD feeding, *n* = 5 mice per group. Data are displayed as mean ± SEM. Significance was calculated by two-way ANOVA with a Bonferroni post-test of multiple comparisons. * indicates *p* ≤ 0.05, ** indicates *p* ≤ 0.01 and *** indicates *p* ≤ 0.001.

Obesity causes hyperinsulinemia, as the pancreatic β cells produce excess insulin in an attempt to compensate for insulin resistance in peripheral tissues [44]. To determine the effects of MnP on hyperinsulinemia, we measured insulin in the sera of mice treated with MnP while on a HFD. Redox modulation by MnP treatment diminished serum insulin levels compared to HFD-fed mice at 5 and 10 weeks of HFD feeding, with a significant reduction seen by 12 weeks (*p* ≤ 0.05) (Figure 5A) indicating lessened hyperinsulinemia and improved utilization of insulin. Leptin is an adipokine hormone involved in regulating food intake and energy metabolism [45] and leptin resistance has been associated with obesity and HFD-feeding, resulting in hyperleptinemia, a decreased satiated feeling [46], and cardiovascular damage. To determine the effects of MnP on hyperleptinemia, leptin was measured in the sera of mice in our model. MnP treatment significantly reduced fasting leptin levels in HFD mice at 5 (*p* ≤ 0.05), 10 (*p* ≤ 0.001), and 12 weeks (*p* < 0.01) (Figure 5B). These observations are consistent with the improved glucose metabolism seen in Figure 3A,B, and with the weight gain reduction (Figure 1), suggesting that lower leptin resistance may preclude polyphagia during early obesity onset.

Obesity induces a series of long-term complications, most notably, an increased cardiovascular risk [47]. PAI-1 is an acute phase protein and primary inhibitor of both tissue-type and urokinase-type plasminogen activators (tPA and uPA)—mediators of fibrinolysis [48,49]. PAI-1 levels are elevated by its production from inflamed liver and adipose tissues [50,51,52]. Therefore, an elevated circulating level of PAI-1 in obesity is considered a risk factor for thrombosis and atherosclerosis [51]. The ability to ameliorate increases in PAI-1 is critical in reducing the cardiovascular risk seen in obesity. Therefore, PAI-1 levels were measured in the sera of mice treated with MnP or control while on a HFD. MnP treatment lowered plasma PAI-1 levels slightly at 10 weeks and significantly (*p* ≤ 0.05) at 12 weeks (Figure 5C). These results demonstrate MnP’s ability to improve obesity-induced cardiovascular risks.

### 3.6. Redox Modulation Minimizes Oxidative Stress-Induced DNA Damage and Pro-Inflammatory Cytokine Production During HFD

**Figure 6:**
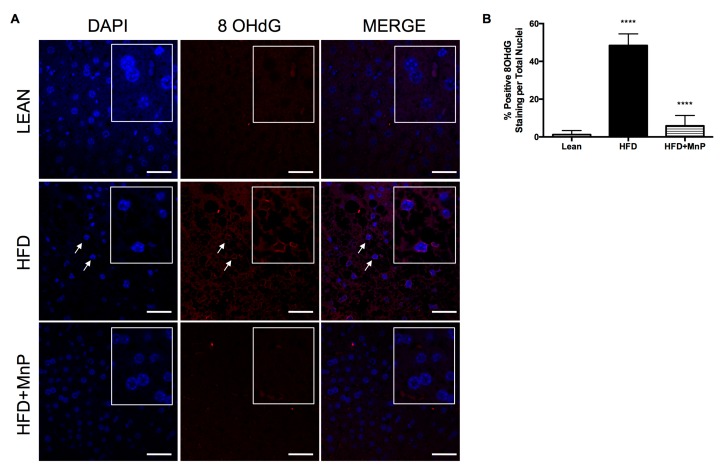
MnP treatment reduces oxidation-induced DNA damage. (**A**) 8-OHdG immunofluorescence staining of livers after 12 weeks of HFD feeding, with or without MnP treatment. Representative images are shown with a 40× magnification. Arrows indicate hepatocyte nuclei positive for 8-OHdG. The inset boxes show a magnified view of the nuclei; (**B**) Quantification of 8-OHdG staining. Data are displayed as mean ± SD, *n* = 3 mice per group. Three images per section were analyzed using Image J Software (National Institute of Health (NIH), Bethesda, MD, USA). Significance was calculated by one-way ANOVA with a Tukey post-test of multiple comparisons. **** indicates *p* ≤ 0.0001 between Lean and HFD, and **** indicates *p* ≤ 0.0001 between HFD and HFD + MnP. Images were created in Photoshop (Adobe Systems Inc., San Joes, CA, USA).

Oxidative stress drives pro-inflammatory cytokine production, which exacerbates NAFLD and T2D pathologies, mediated through oxidation damage to lipids, proteins, and DNA. We have shown with an in vitro model of hepatic steatosis (Figure 1), that MnP quells superoxide levels in response to palmitate stimulation. We next sought to determine whether MnP has similar effects in vivo. To do so, we examined the effect of MnP on oxidative damage at the DNA level in livers of mice treated with MnP. The interaction of the hydroxyl radical with the nucleobase, guanine, forms 8-hydroxy-2′-deoxyguanosine (8-OHdG) [53]. Therefore, the formation of 8-OHdG is therefore a powerful biomarker for the detection of oxidative stress. Indeed, immunofluorescent imaging showed that a HFD significantly increased the levels of oxidative stress-induced DNA damage, by 8-OHdG staining within the nuclei (Figure 6). Strikingly, MnP was able to significantly abolish the presence of 8-OHdG staining in the hepatic nuclei of liver sections, with levels similar to the lean livers (Figure 6). Taken together, MnP was demonstrated to be a potent hepatic antioxidant, both in vivo and in vitro. 

**Figure 7:**
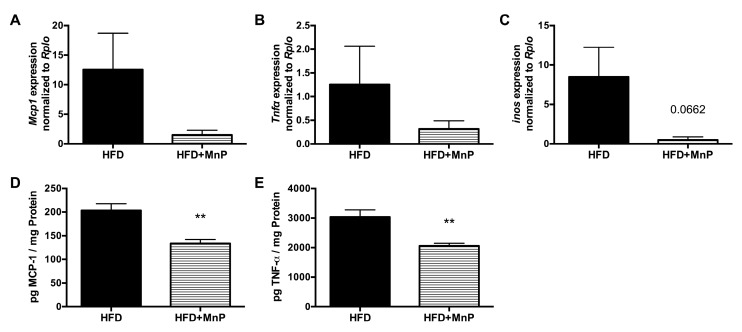
Redox modulation and NF-κB inhibition have anti-inflammation effects in the liver. After 12 weeks of HFD feeding, hepatic gene expression was measured by qRT-PCR for (**A**) MCP1; (**B**) TNFα; and (**C**) inducible nitric oxide synthase (iNOS), with or without MnP treatment. Data are displayed as mean ± SEM, *n* = 5 mice per group. After 12 weeks of HFD feeding, hepatic protein expression was measured by ELISA, for (**D**) MCP-1 and (**E**) TNF-α, with or without MnP treatment. Cytokine concentrations were normalized to protein concentration to yield pg of cytokine per mg of protein. Data are displayed as mean ± SD, *n* = 5 mice per group. Significance was calculated by *t*-tests. ** indicates *p* ≤ 0.01.

The redox-dependent regulation of inflammation is mediated through NF-κB-dependent pro-inflammatory cytokine production. These cytokines are well-accepted mediators of hepatic insulin resistance and are upregulated in obesity [13,14]. Levels of the pro-inflammatory cytokine, TNFα, a potent inhibitor of insulin signaling and therefore a major contributor to insulin resistance, are elevated in obese subjects [54,55]. MCP-1 is a chemokine that aids in this process by recruiting macrophages into liver and adipose tissue, fueling the inflammatory cascade [56,57]. To determine the effects of MnP on NF-κB-mediated cytokine production, the expressions of MCP1 and TNFα were measured in the livers of mice, treated with or without MnP, on HFD. We observed diminished levels of hepatic MCP1 and TNFα gene expressions with MnP treatment (Figure 7A,B). To confirm, we measured the protein levels of these pro-inflammatory mediators from liver tissue and saw significant decreases (*p* ≤ 0.01) in HFD + MnP mice (Figure 7D,E). Previous studies have shown that NF-κB-dependent inducible nitric oxide synthase (iNOS) plays an important role in insulin resistance [58] and that liver-specific inhibition of iNOS reverses obesity-induced insulin resistance [59,60]. Here, we showed diminished levels of hepatic iNOS expression upon redox modulation with MnP (Figure 7C). These data further support the hypothesis that redox modulation by MnP inhibits the NF-κB-dependent production of pro-inflammatory cytokines.

### 3.7. MnP Treatment Inhibits Hepatic Nuclear NF-κB Binding 

**Figure 8:**
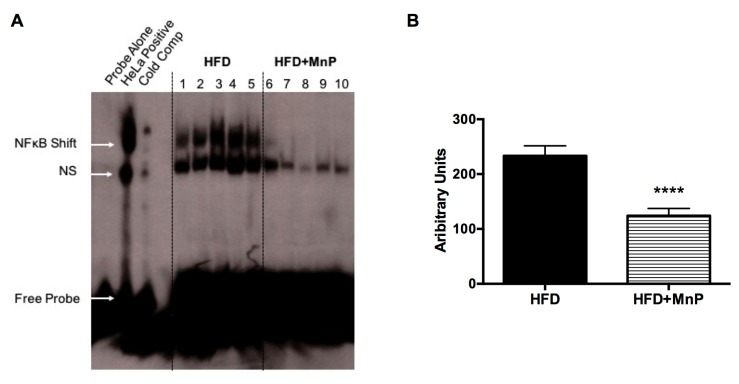
MnP treatment reduces NF-κB binding in the liver. (**A**) After 12 weeks of HFD feeding, nuclear liver extracts from HFD and HFD + MnP mice were mixed with ^32^P-labeled NF-κB consensus oligonucleotide for Electrophoretic Mobility Shift Assay (EMSA). Control samples included ^32^P-labeled NF-κB probe alone, ^32^P-labeled NF-κB probe mixed with HeLa extract, and HeLa extract mixed with 50-fold excess unlabeled NF-κB consensus oligonucleotide. Samples were resolved on a gel. The image displays the NF-κB shift, non-specific binding (NS), and free probe; (**B**) Quantification of NF-κB binding was measured by densitometry. Data are displayed as mean ± SD, *n* = 5 mice per group. Significance was calculated by *t*-tests. **** indicates *p* ≤ 0.0001.

Previously, we demonstrated, in vitro, that MnP prevented the binding of NF-κB to its response element site, more specifically through blocking of NF-κB p50 to its cognate DNA response element [22]. In order to define the mechanism for the anti-inflammatory effects observed with MnP treatment (Figure 7), we determined the degree of NF-κB binding in nuclear liver extracts from HFD fed mice treated, with or without MnP, by EMSA. A significant reduction in hepatic nuclear NF-κB binding was observed with MnP treatment of HFD fed mice (*p* ≤ 0.0001) (Figure 8). These data recapitulate, for the first time in an in vivo model, the blocking of NF-κB DNA binding, correlating with the anti-inflammatory and oxidoreductase mechanism of MnP, previously reported in vitro [22].

## 4. Discussion

During the course of chronic over-nutrition, glycemic control may fail, as a result of insulin resistance. The mechanisms triggering insulin resistance are primarily mediated by oxidative stress and inflammation [36]. Specifically, the liver becomes a sight of abnormal lipid deposition and immune cell infiltrate. Treatment options to reduce the risk of insulin resistance and/or diabetic complications are of major importance. We hypothesized that controlling oxidative stress-induced inflammation by redox modulation and inhibiting hepatic NF-κB activation would assuage NAFLD as well as enhance insulin sensitivity and glucose tolerance.

MnP ameliorates oxidative stress-induced inflammation in immune cells and pancreatic β cells [23,24,25,27,28,37,38,57,61]; however, its role in the liver has not been previously investigated. Here, we used a cell-based experimental system mimicking steatosis. Since HepG2 cells possess NOX3, which is capable of producing superoxide, we anticipated MnP’s efficacy in this model. MnP was able to decrease superoxide production in PA-stimulated HepG2 cells (Figure 1). Therefore, these data set the precedence for using MnP in vivo, using the HFD-induced model of obesity, insulin resistance, and steatosis. In fact, we were able to demonstrate a significant reduction in oxidative DNA damage by MnP, by detection of 8-OHdG in the hepatocyte nuclei of liver sections (Figure 6). Hepatocytes are a prime target in obesity-induced insulin resistance, as the key mediators of glucose metabolism. Additionally, they are able to produce and secrete ROS, resulting in downstream generation of NF-κB-dependent pro-inflammatory cytokines and acute-phase proteins [10,12,13,14,39]—key mediators in insulin resistance. MnP clearly had an effect on hepatocytes, but may also be playing an additional role with the resident and infiltrating hepatic macrophages responsible for driving oxidative stress-induced inflammation. Future studies will elucidate the role of MnP on quelling obesity-induced hepatic macrophage inflammation.

MnP treatment reduced weight gain in HFD-fed mice compared to untreated HFD-fed mice, though HFD + MnP mice gained more weight than the lean mice did (Figure 2). This trend held when comparing the livers of each group. Not only was there a gross difference between the groups upon examining the excised livers (Figure 3A), there also was a significant difference in the weight of livers from HFD + MnP mice, compared to the HFD mice (Figure 3B). This difference in weight can be correlated with the reduced hepatic steatosis (Figure 3C,D) and hepatocellular triglyceride levels following MnP treatment (Figure 3E), suggesting that MnP treatment prevents NAFLD in animals fed a HFD. Notably, these results are not likely attributed to the toxicity of MnP treatment, as demonstrated through assessing multiple diagnostic parameters (Table 1). MnP treatment displayed no organ toxicity, correlating with its already documented safety profile [20].

Further, MnP improved glucose tolerance (Figure 4A) and diminished insulin resistance in HFD-fed animals (Figure 4B,C). These findings point to a potential role for MnP in energy expenditure. We have previously reported that MnP treatment enhances aconitase activity [26]—an enzyme involved in the tricarboxylic acid cycle, which is a direct target of ROS. This would allow for stabilization of the mitochondria leading to more efficient mitochondrial metabolism and improved insulin sensitivity [62]. Reduced serum insulin levels were also observed with MnP treatment, indicating improved insulin signaling and diminished hyperinsulinemia (Figure 5A). These results indicate better glucose utilization, likely caused by decreased inflammatory- and oxidative stress-mediated insulin insensitivity upon MnP treatment. Furthermore, it has been previously described that MnP preserves pancreatic β cell function [37,38]. Therefore, MnP treatment may also be promoting more functional β cells as well as maintaining normal glucose-stimulated insulin secretion, which is generally defective in obesity.

In addition, a significant reduction in serum leptin was observed with MnP treatment (Figure 5B). Leptin is an adipokine that links adiposity and the central nervous system, to reduce appetite and enhance energy expenditure [63,64]. Proper leptin signaling can promote satiety and facilitate glucose utilization, while improving insulin sensitivity [45]. Leptin resistance also occurs from increased caloric intake, and under fasting conditions, elevated serum leptin levels are proportional to the mass of adipose tissue and increase obesity-induced leptin resistance [65] resulting in hyperleptinemia. Indeed, MnP treatment reduced hyperleptinemia contributing to the decreased weight gain, adiposity, and steatosis observed in the MnP treated animals.

Moreover, serum PAI-1 levels were reduced with MnP treatment (Figure 5C). MnP has been shown to repress the expression of PAI-1 in prostate cancer by decreasing transcription factor p300 DNA binding to an hypoxia response element (HRE) motif within the PAI-1 gene promoter region [61]. Therefore, it is not surprising that MnP had effects on PAI-1 in our model. PAI-1 is an acute-phase protein, produced by the endothelium, liver, and adipose tissue [51]. Obesity induces elevated PAI-1 levels concomitant with insulin resistance [50] and hepatic steatosis. Insulin resistance is a result of elevated levels of TNF-α, which also enhances PAI-1 expression [55]; therefore, MnP-mediated reduction in TNF-α and PAI-1 levels synergize to inhibit steatosis and would likely be effective in reducing the risk for thrombosis and atherosclerosis [66].

**Figure 9:**
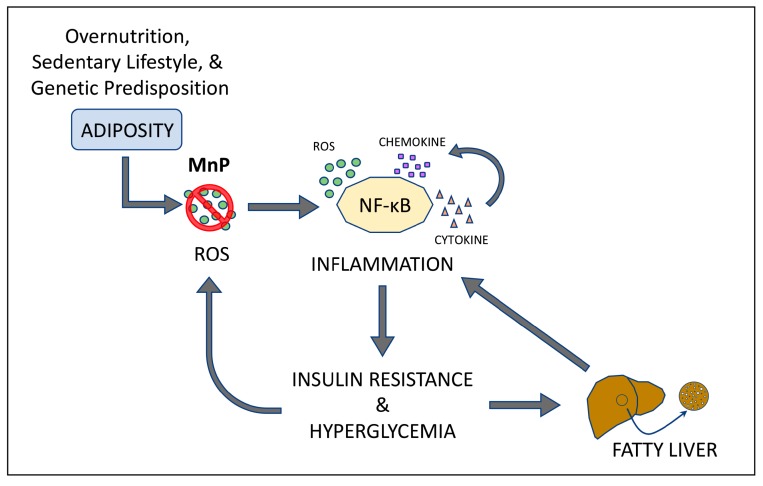
Mechanism of redox modulation by MnP in a HFD model of obesity and insulin resistance. Increased caloric intake, coupled with decreased physical activity along with a genetic predisposition can lead to reactive oxygen species (ROS) production and oxidative stress [2,3,9,10,11]. Uncontrolled obesity-induced oxidative stress results in chronic inflammation and production and circulation of NF-κB-dependent proinflammatory cytokines and chemokines, as well as additional production of ROS [13,14,15], fueling a feedback loop. Inflammation can directly affect insulin signaling, resulting in insulin resistance and hyperglycemia. This not only contributes to the progression of type 2 diabetes (T2D), but also exacerbates ROS production and oxidative stress. Consequently, these events cause hepatic steatosis and subsequent non-alcoholic fatty liver disease (NAFLD), the hepatic manifestation of metabolic syndrome. In addition, there is a perpetuation of insulin resistance, as well as dyslipidemia—hallmarks of T2D progression. Treatment with MnP is known to not only scavenge superoxide, but also possess anti-inflammatory properties [19,21,22,23,24,27,28,29,37,38]. Blockade of nuclear NF-κB activation by MnP was sufficient to diminish pro-inflammatory cytokine production, thereby reducing weight gain, insulin resistance, and hepatic steatosis in HFD-fed mice.

The mechanism behind these striking effects can be attributed to a significant dampening of inflammation (Figure 9). Obesity results in oxidative stress and chronic inflammation, which, in turn, cause insulin resistance and dysregulation of lipid metabolism. Further, the culmination of oxidative stress-induced ROS production leads to activation of the NF-κB pathway, which contributes to NAFLD progression, from simple steatosis to a more inflammatory condition, non-alcoholic steatohepatitis, (NASH) [67]. This suggests that the ability of MnP to combat oxidative stress and inflammation is potentially relevant in the prevention of NAFLD and T2D. Here, we have shown for the first time that blockade of hepatocyte-associated NF-κB activation by MnP (Figure 8) was able to ameliorate the obesity-mediated T2D phenotype, by diminishing the levels of TNF-α, MCP-1, and iNOS (Figure 7A,B), which are major inducers of NASH and insulin resistance [54,56].

A different manganese porphyrin, manganese [19] tetrakis [6]-benzoic acid porphyrin (MnTBAP), has also been investigated for its role in ameliorating the progression of obesity-induced insulin resistance [68,69,70]. However, our study differs in that MnP not only acts as a free radical scavenger [19,21,22,24,26,27,38], but its oxidoreductase activity endows it with powerful anti-inflammatory activity [21,22,25,27]. This is primarily due to the positive charge of this class of MnPs, whereas MnTBAP and other anionic or neutral charged compounds do not prevent NF-κB p50 DNA binding [19]. Although controlling ROS the generation of ROS is important for ameliorating insulin resistance, preventing inflammation is imperative, and both of these linked responses are inhibited by MnP. This is underscored by the fact that MnP treatment reduced the hepatic gene and protein expressions of MCP-1 and TNF-α. MCP-1 recruits macrophages into peripheral sites such as the liver and adipose tissue, which drive the production of TNF-α, described as a potent instigator of obesity-induced insulin resistance [54]. Further, since TNF-α, working synergistically with other cytokines, can activate inducible iNOS expression in the liver [71], it is no surprise that MnP treatment also diminished iNOS expression (Figure 7C).

## 5. Conclusions

In conclusion, this study revealed that inhibiting oxidative stress and NF-κB-mediated inflammation has a marked impact on the progression of T2D, and in doing so, also maintains metabolic homeostasis, thereby improving NAFLD and hepatic insulin resistance in obesity. Future studies are necessary to determine how MnP affects food intake and energy expenditure in mice fed a HFD as well as how it impacts hepatic macrophage recruitment and activation. As such, these findings further elucidate the mechanism of NAFLD in T2D, and point to therapeutic opportunities. 

## Figures and Tables

**Table 1:** MnP treatment exhibits minimal toxicity.

Analyte	Lean	HFD	HFD + MnP	One-Way ANOVA
Albumin (g/dL)	2.617	3.433	4.233	* Lean vs. HFD + MnP
Alanine Aminotransferase (U/L)	63	58.33	79	ns
Alkaline Phosphatase (U/L)	55.17	54.5	54	ns
Amylase (U/L)	921.5	1042	1092	ns
Blood Urea Nitrogen (mg/dL)	23.17	19.67	22.5	ns
Calcium (mg/dL)	10.5	10.18	10.32	ns
Creatinine (mg/dL)	0.1667	0.2	0.2	ns
Globulin (g/dL)	2.167	2.317	1.85	ns
Glucose (mg/dL)	121.5	141.5	142.2	ns
Phosphorus (mg/dL)	8.75	8.083	7.833	ns
Potassium (mmol/L)	5.583	5.083	4.633	ns
Sodium (mmol/L)	151.5	150.8	152.5	ns
Total Bilirubin (mg/dL)	0.1833	0.2333	0.2333	ns
Total Serum Protein (g/dL)	5.817	5.733	6.067	ns

MnP treatment exhibits minimal toxicity, *n* = 5 mice per group. Significance was calculated based on mean and SD by one-way ANOVA, with a Tukey post-test of multiple comparisons. * indicates *p* ≤ 0.05.
